# Photoresponsive immunomagnetic nanocarrier for capture and release of rare circulating tumor cells†
†Electronic supplementary information (ESI) available: Detailed experimental procedures and materials; Fig. S1–S8 and Tables S1–S3. See DOI: 10.1039/c5sc01380a


**DOI:** 10.1039/c5sc01380a

**Published:** 2015-07-30

**Authors:** Song-Wei Lv, Jing Wang, Min Xie, Ning-Ning Lu, Zhen Li, Xue-Wei Yan, Si-Liang Cai, Ping-An Zhang, Wei-Guo Dong, Wei-Hua Huang

**Affiliations:** a Key Laboratory of Analytical Chemistry for Biology and Medicine (Ministry of Education) , College of Chemistry and Molecular Sciences , Wuhan University , Wuhan 430072 , China . Email: whhuang@whu.edu.cn ; Fax: +86-27-68754067 ; Tel: +86-27-68752149; b Department of Gastroenterology , Renmin Hospital of Wuhan University , Wuhan 430060 , China . Email: dwg@whu.edu.cn; c Department of Clinical Laboratory , Renmin Hospital of Wuhan University , Wuhan 430060 , China

## Abstract

Isolation, release and culture of rare circulating tumor cells (CTCs) may, if implemented, promote the progress of individualized anti-tumor therapies. To realize the release of CTCs without disruption of their viability for further culture and analysis, we designed an effective photocontrolled CTC capture/release system by combination of photochemistry and immunomagnetic separation. 7-Aminocoumarin was synthesized as the phototrigger to bridge the connection between the anti-EpCAM antibody and the magnetic beads. The coumarin moieties produced cleavage of a C–O bond under both ultraviolet (UV) and near-infrared (NIR) light illumination, breaking the bridge and releasing CTCs from the immunomagnetic beads. Compared with conventional immunomagnetic separation systems, the negative influence of absorbed immunomagnetic beads on further CTCs culture and analysis was effectively eliminated. The system can specifically recognize 10^2^ MCF-7 cells in 1 mL of human whole blood samples with 90% efficiency and 85% purity. Under the irradiation of UV and NIR light, 73 ± 4% and 52 ± 6% of captured cells were released with a viability of 90% and 97%, respectively. Furthermore, this technique has been used to detect CTCs from whole blood of cancer patients with high purity. This study demonstrates that the photochemical-based immunomagnetic separation method for isolating, releasing and culturing CTCs from clinic patients may provide new opportunities for cancer diagnosis and personalized therapy.

## Abstract 2

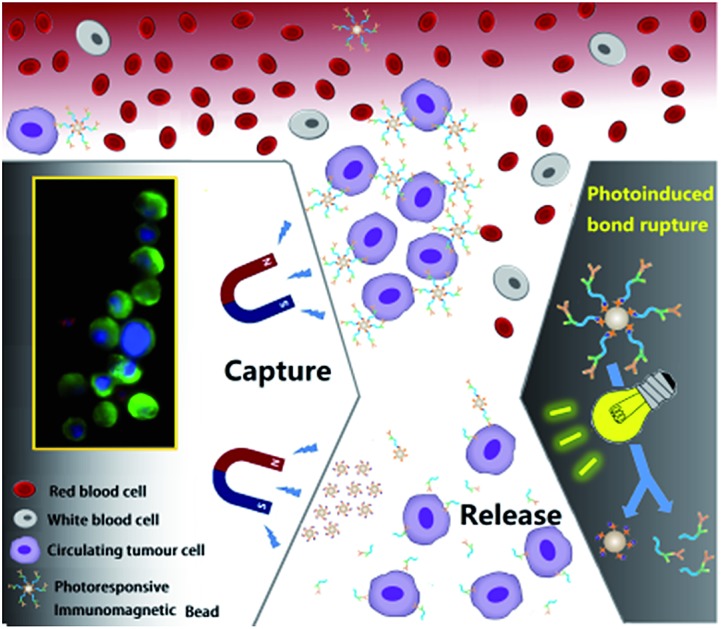
7-Aminocoumarin compound was synthesized and used as phototrigger to cage EpCAM-antibody to construct a photocontrolled CTCs capture and release system.

## Introduction

Metastasis is the cause of most cancer deaths in patients with solid tumors.^1^,^2^ Circulating tumor cells (CTCs) are cells released from the primary tumor into the bloodstream that are considered the main promoters of metastasis.^3^,^4^ Compared to biopsy (the gold standard of current cancer diagnosis), CTC detection offers convenient and non-invasive access to tumor cells before fatal metastasis occurs.^5^,^6^ To exploit CTCs as a “liquid biopsy” for disease progression and guide implementation of therapy, over the past decade, many techniques have been developed for CTC isolation and enrichment, for instance, flow cytometry,^7^,^8^ microfluidic chips,^9^–^11^ immunomagnetic separation,^12^–^15^ and CTC filters.^16^,^17^ Among them, magnetic separation is a promising tool for CTC enrichment, because of its easy modification, fast magnetic response and high capture efficiency. The current FDA cleared CellSearch Assay^18^,^19^ is also based on immunomagnetic separation of CTCs and shows good stability and reproducibility for CTC detection.

The present-day CTC detection methods focus not only on the capture of CTCs from patients, but also on subsequent culture and analysis, since further independent study in the CTCs isolated from patient samples can provide additional information that leads to progress in individualized anti-tumor therapies. However, CTCs are usually captured and adhere tightly on the substrates of capture platforms, and must be released from these substrates for further culture and analysis. Although magnetic beads (MBs)-based techniques can isolate individual CTCs from whole blood, the adsorption of numerous magnetic nanoparticles on cells leads to severely negative influences for further analysis such as inhibition of cell re-culture and distortion effects on accurate image analysis.^14^,^20^–^23^ Therefore, releasing the captured CTCs from the carrier surface becomes a very important and challenging step. Methods like thermodynamic release,^24^–^26^ chemical competitive combination triggered release,^14^,^27^,^28^ electrochemical desorption^29^–^31^ and proteolytic enzyme degradation^10^,^20^ have been used to release captured tumor cells. However, the majority of these methods are invasive, with the potential to harm the completeness of cell structure and disturb the cell microenvironment.

Recently, photocontrolled release systems based on light-induced bond cleavage or structural changes have attracted much attention for their applications in the area of drug/gene delivery^32^–^38^ and photoswitched cell adhesion.^39^–^41^ Photocontrolled release systems are non-invasive to the biological system and possess the possibility of remote spatiotemporal control. Cell release can be controlled precisely by external manipulation, through changing the irradiation parameters such as wavelength, intensity and time, providing the possibility for site-specific cell release.^42^ However, applying photocontrolled systems to CTC release has hitherto rarely been reported.^43^,^44^

**Scheme 1:**
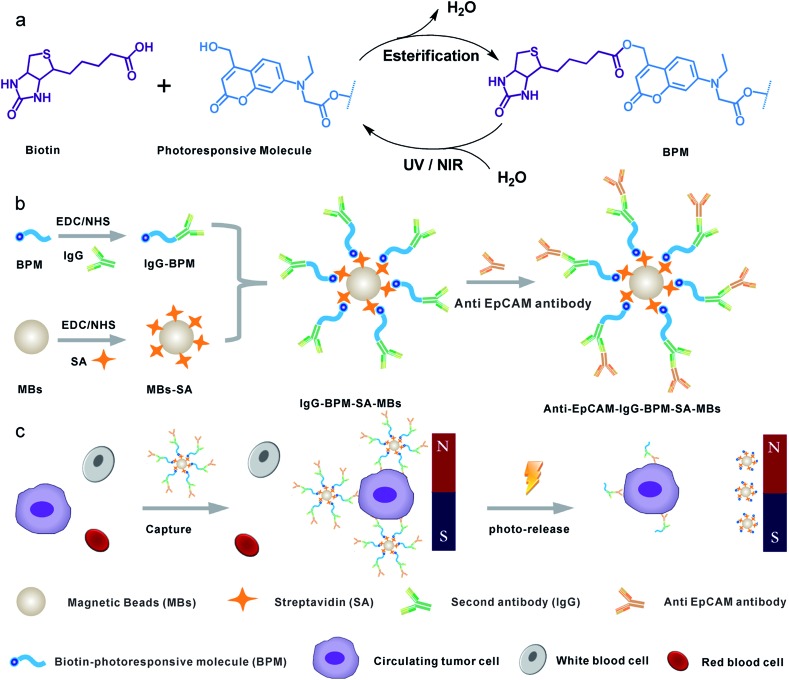
Schematic diagram showing the photoresponsive immunomagnetic system for capture and release of CTCs. (a) Synthesis and photo-induced cleavage of biotin-photoresponsive molecule (BPM); (b) construction of photoresponsive immunomagnetic beads; (c) capture and photo-induced release of CTCs.

Herein, we constructed a novel CTC capture and release system by combination of photochemistry and immunomagnetic separation. 7-Aminocoumarin was synthesized, and reacted with biotin to form a photoresponsive linker (Scheme 1a). This photoresponsive linker was then used to bridge the capture antibody and streptavidin (SA) modified MBs (magnetic hysteresis loop and time-dependent magnetic separation efficiency are shown in Fig. S1†) (Scheme 1b). Thus the whole system constructed as antibody–photoresponsive linker–magnetic beads fulfils three functions: specific capture, magnetic separation and photo-release. After CTC capture, upon the application of a non-invasive UV or NIR light irradiation, the coumarinylmethyl moieties produced cleavage of a C–O bond^45^,^46^ (Scheme 1a), which realized the release of the immunomagnetic immobilized CTCs (Scheme 1c). 73 ± 4% and 52 ± 6% of captured cells were released under the UV and NIR light irradiation with a viability of 90% and 97%, respectively. This strategy effectively eliminates the optical distortion effect of beads and ensures accurate image analysis for CTCs; more importantly, CTCs were relieved from the side-effects created by the presence of adsorbed beads, promoting further cell re-culture. Furthermore, this system has been used to detect CTCs from whole blood of cancer patients with high purity, indicating that the photochemical-based immunomagnetic separation method may provide new opportunities for cancer diagnosis and personalized therapy.

## Results and discussion

### Synthesis of photoresponsive linker

**Fig. 1:**
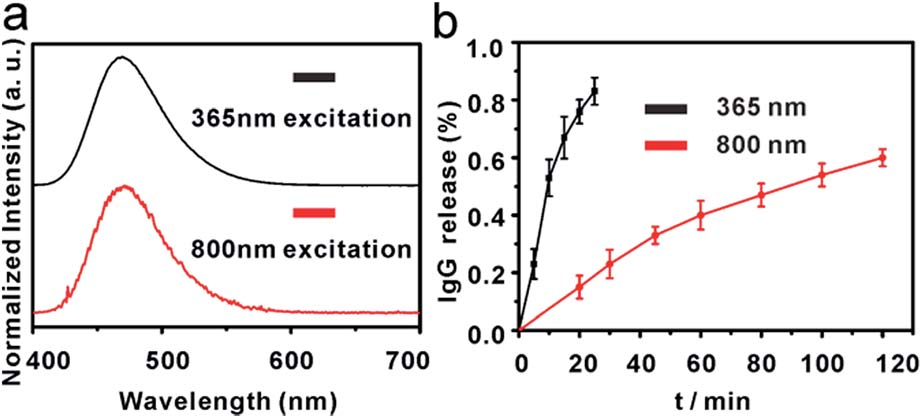
(a) Emission spectra of compound 7 (upper: single photon excitation at 365 nm, lower: two photon excitation at 800 nm); (b) time course of photolysis controlled IgG release from IgG-BPM-SA-MBs (left: 365 nm, 10 mW cm^–2^; right: 800 nm, 10 mW cm^–2^) (error bars represent standard deviations, *n* = 3).

In this work, a small molecule, a 7-aminocoumarin compound, was chosen and synthesized as the core part to construct a photoresponsive linker, due to its excellent properties in photo-response. The coumarin photo-cleavable groups have high molar extinction coefficient, rapid photolysis rate, NIR excitation and low toxicity of photolysis side-products for biological systems.^47^–^49^ The coumarin moieties produce cleavage of a C–O bond under illumination, leading to a separation of the two ends. Furthermore, as a synthetic product, 7-aminocoumarin derivatives can be designed with various functional groups to fit different application systems. Here, a 7-aminocoumarin compound with a hydroxyl group and a carboxyl group was reacted with biotin *via* the hydroxyl group to construct a biotin-7-aminocoumarin compound (biotin-photoresponsive molecule, BPM) as the photoresponsive linker, and the carboxyl group was subsequently used for conjugation with antibodies (Fig. S2†). Fluorescence spectra were recorded by using a single- and a two-photo excitation light source to test the two-photon absorption properties of the photoresponsive linker. The results showed that the linker possessed both single- and two-photon fluorescence properties (Fig. 1a).

### Fabrication of IgG-BPM-SA-MBs

Scheme 1 illustrates the entire construction process of photoresponsive immunomagnetic beads and the photo-induced release of cells from MBs. To construct a photoresponsive system, SA-immobilized MBs (SA-MBs) (Fig. S3†) and BPM caged IgG (IgG-BPM) were prepared, and IgG-BPM-SA-MBs were then obtained by the specific interaction between SA and biotin. The successful preparation of IgG-BPM-SA-MBs was evaluated by fluorescent labelling (Fig. S4†) as well as particle size analysis (Fig. S5†). Here, SA-biotin was employed to mediate the connection of phototrigger caged antibody and MBs which avoided the direct interaction between antibody and MBs, guaranteeing the high photo-induced release efficiency. To determine the optical exposure time for bond cleavage, the time courses of the IgG release under photolysis at both 365 nm light and 800 nm NIR light (Fig. 1b) were monitored by UV-visible spectra. Both photolytic processes progressed effectively and the release controlled by 365 nm (10 mW cm^–2^) irradiation reached 75% after 15 min while 800 nm (10 mW cm^–2^) irradiation reached 60% after 2 h.

### Fabrication and characterization of anti-EpCAM-IgG-BPM-SA-MBs

**Fig. 2:**
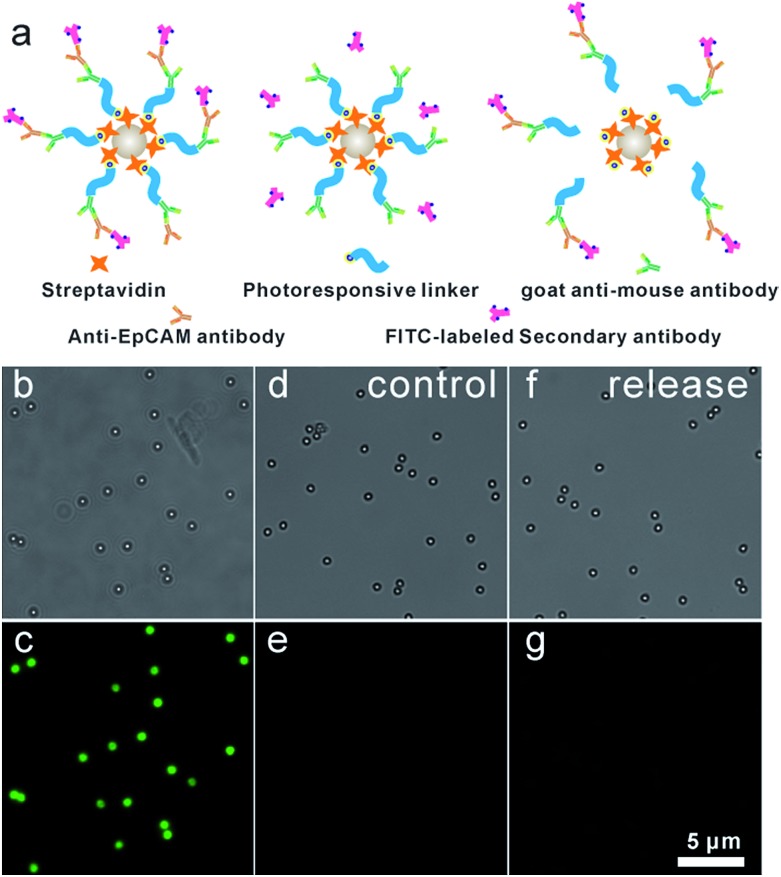
7-Aminocoumarin enabled conjugation and release of anti-EpCAM on IgG-BPM-SA-MBs. (a) Schematic graph showing the principle of confirming conjugation of anti-EpCAM on IgG-BPM-SA-MBs; (b and c) incubation of FITC-labelled secondary antibody with anti-EpCAM-IgG-BPM-SA-MBs; (d and e) incubation of FITC-labelled secondary antibody with IgG-BPM-SA-MBs; (f and g) after light irradiation (365 nm, 10 mW cm^–2^, 15 min) to anti-EpCAM-IgG-BPM-SA-MBs, the remaining MBs part reported by FITC-labelled secondary antibody (panels b, d, and f show bright-field images; panels c, e, and g show fluorescence images).

Anti-EpCAM was immobilized onto IgG-BPM-SA-MBs through specific recognition between a secondary antibody and a primary antibody to construct anti-EpCAM-IgG-BPM-SA-MBs. Subsequently, a FITC-labelled secondary antibody (Fig. 2) was used to report the attachment and light-induced detachment of anti-EpCAM onto/from the surface of IgG-BPM-SA-MBs. Compared to the faint fluorescence of IgG-BPM-SA-MBs (Fig. 2e), anti-EpCAM-IgG-BPM-SA-MBs showed strong FITC fluorescence (Fig. 2c). Upon light irradiation treatment, the 7-aminocoumarin in BPM produced a cleavage of the ester bond, leading to the release of anti-EpCAM-IgG-BPM from the MB surface, while the remaining MB part could not be labelled by the FITC-labelled secondary antibody (Fig. 2g).

### Capture and release of cancer cells using anti-EpCAM-IgG-BPM-SA-MBs

**Fig. 3:**
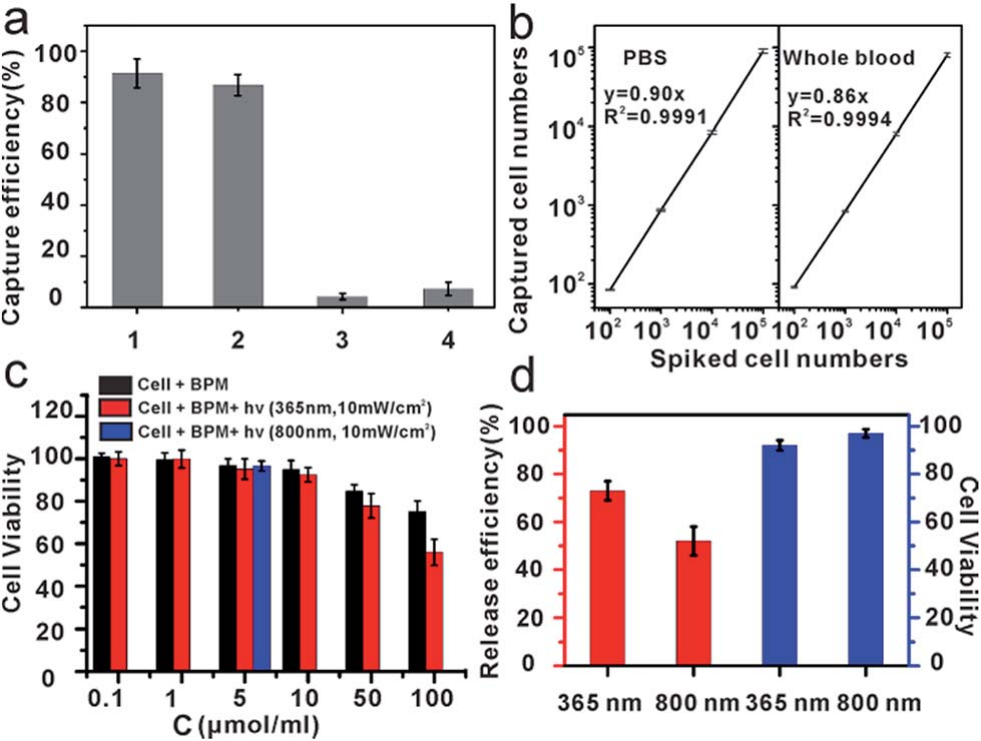
Capture and release of cancer cell. (a) Capture efficiencies of cancer cells. 1: anti-EpCAM-IgG-BPM-SA-MBs to MCF-7 cells; 2: anti-EpCAM-IgG-BPM-SA-MBs to SK-BR-3 cells; 3: anti-EpCAM-IgG-BPM-SA-MBs to HeLa cells; 4: IgG-BPM-SA-MBs to MCF-7 cells; (b) regression analysis of the number of the MCF-7 cells captured by the anti-EpCAM-IgG-BPM-SA-MBs *versus* the number of the cells spiked in two different types of samples: left PBS, right whole blood (error bars represent standard deviation, *n* = 3); (c) cytotoxicity assay: effect of photosensitive molecule with different concentrations on the cell viability of MCF-7 cell line, the light exposure time is 15 min (*λ* = 365 nm, 10 mW cm^–2^) and 2 h (*λ* = 800 nm, 10 mW cm^–2^); (d) release efficiencies of captured MCF-7 cells by light irradiation (left: 365 nm, 10 mW cm^–2^; right: 800 nm, 10 mW cm^–2^), and cell viability after release by different wavelength of light (left: 365 nm, right: 800 nm).

To explore the specific cell recognition performance of anti-EpCAM-IgG-BPM-SA-MBs (Fig. 3a), two EpCAM-positive cancer cell lines (MCF-7, SK-BR-3) were chosen as the target cell lines,^50^ and HeLa (EpCAM-negative cancer cell lines)^51^ was selected as control. EpCAM is frequently over-expressed by many kinds of solid-cancer cells and is absent from hematologic cells.^52^ Compared with the EpCAM-negative cells (HeLa), the anti-EpCAM positive cells (MCF-7 and SK-BR-3 cells) display high specific cell capture efficiency through anti-EpCAM-IgG-BPM-SA-MBs (MCF-7, 91 ± 5%; SK-BR-3, 87 ± 4%). Meanwhile, the IgG-BPM-SA-MBs could hardly capture MCF-7 cells (Fig. 3a), indicating that the binding between anti-EpCAM-IgG-BPM-SA-MBs and MCF-7 cells was effective and specific. The capability of anti-EpCAM-IgG-BPM-SA-MBs to capture rare tumor cells in synthetic CTC samples was investigated. DAPI-stained MCF-7 cells were spiked into whole blood with a concentration of 10^2^, 10^3^, 10^4^, 10^5^ cells mL^–1^; for comparison, capture efficiencies were also examined in PBS buffer spiked with similar concentrations of MCF-7 cells. As shown in Fig. 3b, regression analysis of captured cell number *versus* spiked cell number gave *y* = 0.90*x* (*R*^2^ = 0.9991), *y* = 0.86*x* (*R*^2^ = 0.9994), respectively in PBS and whole blood. It can be seen that anti-EpCAM-IgG-BPM-SA-MBs could specifically and efficiently capture target cells.

### Toxicity of materials and the viability of released cells

To study the effect of photosensitive molecules and optical radiation on cell viability, the cytotoxicity of materials was determined by MTT method in breast cancer cell line MCF-7. As demonstrated in Fig. 3c, with a concentration of photoresponsive molecule of 0.1 to 10 μmol ml^–1^, the MCF-7 cells did not lose their viability or show detectable changes in behavior after 24 hours of incubation, which demonstrated that the synthesized material was of good biocompatibility.

According to the results of photo-release of IgG-BPM from SA-MBs (Fig. 1) and the previously optimized conditions for photo-cleavage of coumarin,^53^,^54^ cell release was carried out under two different wavelengths of radiation. Before cell capture, there were large populations of MCF-7 cells on the 96-well cell-culture plate (Fig. S6a†). After incubation with anti-EpCAM-IgG-BPM-SA-MBs for 30 min and magnetic scaffold separation (in the dark), few cells remained on the 96-well cell-culture plate (Fig. S6b†). When the light irradiation (365 nm, 10 mW cm^–2^) was applied for 15 min, an average of 73 ± 4% of the captured MCF-7 cells were released from the anti-EpCAM-IgG-BPM-SA-MBs (Fig. 3d and S6c†), and an average of 52 ± 6% of the captured cells were released under NIR light irradiation (800 nm, 10 mW cm^–2^, 2 h) (Fig. 3d and S6d†). Meanwhile, the released cancer cells can be directly cultured and propagated *in vitro* (Fig. S7†).

Calcein AM and propidium iodide (PI) were used to stain the released tumor cells to analyze their viability. Calcein AM can penetrate the live cell membrane and react with the intracellular esterase to form calcein with green fluorescence, while PI is a membrane-impermeable nuclear stain that can stain only dead cells, resulting in red fluorescence.^55^,^56^ From Fig. 3d and S8,† it can be seen that the majority of the released cells showed green fluorescence, and the viability rate was calculated to be 90% (365 nm) and 97% (800 nm). The results showed that released cells maintained a good viability under irradiation at both 365 nm and 800 nm, while 365 nm irradiation induced a faster and more efficient cell release performance. Cell viability under different UV-irradiation times was further tested. The results showed that cell viability was obviously sacrificed with elongated irradiation time (Table S1†), though increasing irradiation time improved the release efficiency (Fig. 1b). Therefore, we finally selected a UV-irradiation time of 15 min in our following photo-induced releasing experiments, which maintained a good balance between release efficiency and cell viability. With this approach, the anti-EpCAM-IgG-BPM-SA-MBs on the cancer cell surface could be rapidly and efficiently released without damaging the cells, which was crucial for CTC cellular analysis.

### Capture and ICC identification of spiked cancer cells from mimic clinical blood samples

**Fig. 4:**
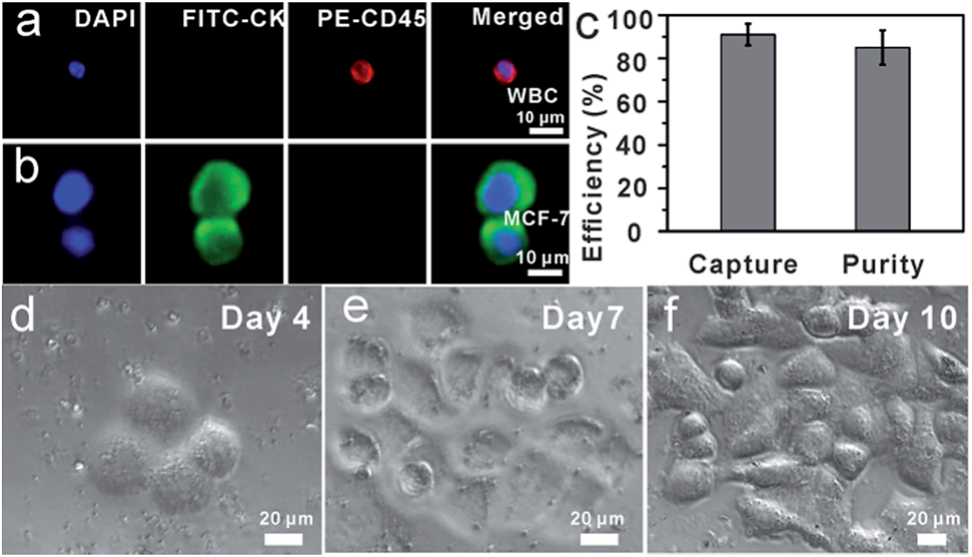
Capture of MCF-7 cells from whole blood. (a and b) Microscopic images of cells captured from mimic clinical blood samples and identified with the three-color ICC. Merged: merge of nucleus (DAPI), CK (FITC), and CD45 (PE); (c) capture efficiency of MCF-7 in the whole blood and purity; (d–f) re-culture of released cancer cells from mimic clinical blood samples (5 × 10^2^ MCF-7 cells were spiked into 1 mL whole blood) (d) Day 4, (e) Day 7, (f) Day 10.

To demonstrate the isolation of cancer cells from human whole blood, DAPI-stained MCF-7 cells were spiked into healthy human whole blood with a concentration of approximately 10^2^ cells mL^–1^. As shown in Fig. 4, as few as 10^2^ MCF-7 cells were effectively isolated and detected from 1 mL mimic patient blood with 90% ± 5% capture efficiency (*n* = 3), demonstrating that anti-EpCAM-IgG-BPM-SA-MBs were applicable in the isolation and detection of rare CTCs. Further, the captured cancer cells could be used for common three-color immunocytochemistry (ICC) identification by FITC-labelled anti-CK19 (a marker for epithelial cells) monoclonal antibody, PE-labelled anti-CD45 (a marker for WBCs), and DAPI nuclear staining. As shown in Fig. 4a–b, the MCF-7 cell was DAPI+/CK+/CD45– and WBCs were DAPI+/CK–/CD45+. Meanwhile, we measured that the purity of separated MCF-7 cells was 85% ± 8% (Fig. 4c). These results showed that cancer cells can be isolated by anti-EpCAM-IgG-BPM-SA-MBs from whole blood.

To determine whether the released tumor cells can be cultured, 5 × 10^2^ MCF-7 cells were spiked into 1 mL whole blood and subjected to the capture and release process as discussed above. The released cells were then seeded into cell culture dishes for propagation in culture (Fig. 4(d–f)). Compared with the control group, re-culture of the released cells showed the same viability, which might have great potential for the subsequent molecular and functional analysis.

### Isolation of CTCs from cancer patient blood samples

**Fig. 5:**
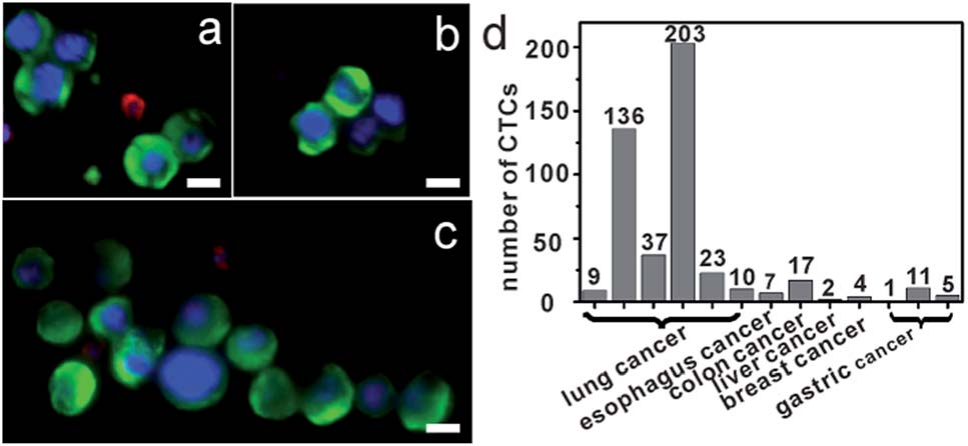
Capture of CTCs from whole blood of cancer patients. (a–c) Micrographs of CTCs (a) and CTCs clusters (b and c) isolated from a metastatic lung cancer patient based on anti-EpCAM-IgG-BPM-SA-MBs, immunofluorescence staining (DNA (blue), CK (green), and CD45 (red)); (d) quantification of CTCs of blood samples from patients. All scale bars represent 10 μm.

Further, we applied anti-EpCAM-IgG-BPM-SA-MBs to the detection of CTCs in the whole blood samples from 13 cancer patient samples (including colon, liver, lung, and breast cancer patients). We also processed blood from healthy individuals as control (*n* = 8). The isolated cells were also identified with the three-color ICC as described above, and CTCs were DAPI+/CK+/CD45–, and WBCs were DAPI+/CK–/CD45+. Images of CTCs captured with our photoresponsive immunomagnetic beads from 1.5 mL of blood from patient #4 are shown in Fig. 5a–c and the results are summarized in Fig. 5d and Tables S2 and S3 (ESI†). CTCs in the blood of the 13 cancer patients could be captured and detected, while no CTCs were found in any healthy samples.

An important finding of this study is the successful isolation of CTC clusters from the blood of a patient with metastatic lung cancer. Although there is evidence to show that, compared with single CTCs, CTC clusters have 23- to 50-fold increased metastatic potential, the presence and biological significance of such CTC clusters in blood are still not well understood.^57^,^58^ The successful capture of CTC clusters in the blood of patients with cancer may provide insight into the process of metastasis in human cancer. Further research for these clusters will provide more possibilities to clarify the mechanism of tumor metastasis.

## Conclusions

In conclusion, we developed a strategy for isolating and releasing CTCs using biotin-7-aminocoumarin as phototrigger to cage anti-EpCAM antibody to constitute photoresponsive immunomagnetic system. This system can not only isolate CTCs with high specificity, but also releases CTCs without disruption of its viability and biological functions. The anti-EpCAM-IgG-BPM-SA-MBs could specifically recognize 10^2^ MCF-7 cells in 1 mL of human whole blood sample with 90% efficiency and 85% purity. Under UV and NIR light irradiation, 73 ± 4% and 52 ± 6% of captured cells were released from MBs with the viability of 90% and 97%, respectively. The released cells maintained the ability to proliferate, which is a critical requirement for personalized medicine. Furthermore, the photoresponsive immunomagnetic beads were applied to clinic CTC detection, including isolation of individual CTCs or CTC clusters from metastatic cancer patients and characterization based on three-color ICC method. Therefore, our CTC capture and release system shows great potential for efficient CTC enrichment, isolation and culture. Our future efforts will include releasing and culturing the captured CTCs from cancer patients, as well as cellular and genetic analysis of the isolated CTCs.

## Supplementary Material

Supplementary information
