# Supplementary material for: Photoresponsive immunomagnetic nanocarrier for capture and release of rare circulating tumor cells
Source: Chem Sci. 2015 Jul 30;6(11):6432–8. doi: 10.1039/c5sc01380a (PMC5507187; doi:10.1039/c5sc01380a)
Supplement: Supplementary file 1 [file SC-006-C5SC01380A-s001.pdf]

Electronic Supplementary Material (ESI) for Chemical Science.
This journal is © The Royal Society of Chemistry 2015

Supporting Information for Photoresponsive Immunomagnetic Nanocarrier for Capture and Release of Rare Circulating Tumor Cells

*Song-Wei Lv,[a †] Jing Wang,[b †] Min Xie,[a] Ning-Ning Lu,[a] Zhen Li,[a] Xue-Wei Yan,[a] Si-Liang Cai,[a] Ping-An Zhang,[c] Wei-Guo Dong[b] * and Wei-Hua Huang[a] **

a. Key Laboratory of Analytical Chemistry for Biology and Medicine (Ministry of Education), College of Chemistry and Molecular Sciences, Wuhan University, Wuhan 430072, China. E-mail: whhuang@whu.edu.cn; Fax: +86-27-68754067; Tel: +86-27-68752149.

b. Department of gastroenterology, Renmin Hospital of Wuhan University, Wuhan 430060, China. E-mail: dwg@whu.edu.cn.

c. Department of Clinical Laboratory, Renmin Hospital of Wuhan University, Wuhan 430060, China.

METHODS

Materials and Instruments

All starting materials were analytical grade from commercial sources and used without further purification. Acetonitrile, dichloromethane, N, N-Dimethylformamide (DMF), p-xylene were freshly distilled before use. Dynabeads® MyOneTM Carboxylic Acid were purchased from Invitrogen (binding capacity: 500 μg / ml beads; bead size: 1 μm; ζ-potential: -36.0 ± 1 mV). Goat anti-mouse IgG, FITC-labeled rabbit anti-goat secondary antibody were provided from Boster Company. Streptavidin, biotin (5-fluorescein) conjugate (FITC-biotin), biotin, 1-ethyl-3-(3-(dimethylamino)propyl) carbodiimide hydrochloride (EDC), N-hydroxysuccinimide (NHS), anti-EpCAM mouse monoclonal antibody, FITC-labeled goat anti-mouse secondary antibody, 4',6-diamidino-2-phenylindole (DAPI), Calcein-AM, and propidium iodide (PI) were purchased from Sigma-Aldrich. FITC-labeled mouse anti-human anticytokeratin (FITC-CK) and PE-labeled mouse anti-human CD45 (PE-CD45) were obtained from Abcam Company. All other chemicals were supplied by Shanghai Chemical Reagent Company.

Apparatus and Characterization:

^{1}H and ^{13}C NMR and spectra were measured on a Varian Mercury 300 spectrometer using tetramethylsilane (TMS; δ = 0 ppm) as internal standard. Mass spectra were recorded on a Brucker Daltonics APE XII 47e. UV-visible spectra were obtained using a Shimadzu UV-2550 spectrometer. Fluorescence microscopic images were obtained using a Zeiss microscope (AxioObserver Z1, Zeiss, Germany). Magnetic hysteresis loops were measured with a vibrating sample magnetometer. Particle size analysis was obtained with Zetasizer Nano, Malvern. The NIR

light release were obtained by exciting the photoresponsive molecule with a titanium-sapphire laser source (Coherent Inc, Mira 900, 200 fs) set at wavelength 800 nm.

Synthesis of Photo-responsive Linker. The synthetic route is presented in supporting information (Figure S2). Briefly, the basic 7-Amino coumarin molecule compound 3 (Figure S2) was synthesized from 3-aminophenol, through nucleophilic substitution reaction and cyclization reaction, then functionalized into compound 5 (Figure S2) with a -OH group. Compound 5 react with biotin to get photoresponsive linker biotin-7-Amino coumarin (compound 6, Figure S2). The photoresponsive linker was further reacted into compound 7 with -COOH group by removal of tertiary butyl group, in order to conjugate with capture antibodies. The target molecule of each step was characterized by ^{1}H-NMR, ^{13}C-NMR, and mass spectra method.

Fabrication and Characterization of IgG-BPM-SA-MBs. The fabrication of IgG-BPM-SA-MBs is illustrated in Scheme 1b. Firstly, the IgG-BPM was constructed by linking the goat anti-mouse (IgG) antibody with the photoresponsive linker (compound 7, BPM). Secondly, carbodiimide chemistry was used to cross-link amines of the SA with the carboxylic acid groups on the surface of MBs. Approximately 2 mg of MBs was activated in 50 mM EDC and 50 mM NHS in 1 mL of ultrapure water at room temperature with gentle shaking for 30 min. After that, the activated MBs were separated by a magnetic scaffold and washed with ultrapure water three times. Then, they were resuspended in 1 mL of ultrapure water to react with 400 μg of SA for about 2 h with continuous shaking at room temperature. The resultant MBs-SA were washed with ultrapure water to remove surplus SA and then stored in ultrapure water at 4 °C for use. FITC-biotin was used to confirm whether or not SA successfully conjugated onto the surface of MBs. Simply, 50 μL of 5 mg / mL SA-MBs or MBs were incubated with 2 μL of 50 μg / mL FITC-biotin for 30 min,

respectively. After incubation, the samples were washed three times and finally dispersed in ultrapure water for microscopic observation. Finally, 0.2 mg MBs-SA was resuspended in 1 mL of ultrapure water to react with 5 μM of IgG-BPM for about 2 h with continuous shaking at room temperature (in the dark condition). 2 μL of 100 μg / mL FITC-labelled rabbit anti-goat secondary antibody was used to incubate with 5 μL of 5 mg / mL IgG-BPM-SA-MBs or SA-MBs for 30 min (in the dark condition), respectively. After removal of excess FITC-labelled rabbit anti-goat secondary antibody, the reconstituted IgG-BPM-SA-MBs or SA-MBs were observed under fluorescent microscope.

Fabrication and Characterization of Anti-EpCAM-IgG-BPM-SA-MBs. 2 μL of 100 μg / mL FITC-labelled secondary antibody was used to incubate with 50 μL of 5 mg / mL anti-EpCAM-IgG-BPM-SA-MBs or IgG-BPM-SA-MBs for 30 min (in the dark condition), respectively. After removal of excess FITC-labelled secondary antibody, the reconstituted anti-EpCAM-IgG-BPM-SA-MBs or IgG-BPM-SA-MBs were observed under fluorescent microscope. Meanwhile, after light irradiation (365 nm, 10 mW/cm^2, 15 min) to anti-EpCAM-IgG-BPM-SA-MBs, the remaining MBs part showed none fluorescent when labelled by the FITC-secondary, followed by microscopic observation.

Cell Culture. Two breast cancer cell lines (MCF-7, SK-BR-3) and one cervical cancer cell line (Hela) cancer cell lines were obtained from China Center for Type Culture Collection. All cell culture media and supplements were obtained from Gibco Corp. MCF-7, SK-BR-3 and Hela cells were grown in Dulbecco's Modified Eagle Medium (DMEM). All media were supplemented with 10 % fetal bovine serum (FBS) and 1 % Penicillin-Streptomycin. Cells were grown at 37 °C and 5 % CO_2 (HERACELL 150i, Thermo Scientific). Cells were harvested using accutase to ensure that surface

antigens are not degraded by prolonged exposure to more traditional reagents, such as Trypsin-EDTA.

Capture, Release and Culture of Cancer Cells Using Anti-EpCAM-IgG-BPM-SA-MBs. Hemocytometry was taken to examine the capture and release efficiency of anti-EpCAM-IgG-BPM-SA-MBs to MCF-7 cells. MCF-7 cells cultured in a flask were detached and collected as mentioned above. Subsequently, the cells were washed and resuspended in 1 × PBS, and the cell concentration was determined by hemocytometry. Anti-EpCAM-IgG-BPM-SA-MBs were used to capture MCF-7 cells (1.0×10^5 cells per mL). As controls, anti-EpCAM-IgG-BPM-SA-MBs were used to treat Hela cells and SK-BR-3 cells, IgG-BPM-SA-MBs were used to treat MCF-7 cells to investigate the specificity. Then, 50 μL of 5 mg / mL anti-EpCAM-IgG-BPM-SA-MBs were mixed with 10^2, 10^3, 10^4, 10^5 MCF-7 cells in 200 μL of whole blood or PBS respectively, incubated for 30 min at room temperature (in the dark condition), and then separated with a magnetic scaffold for 5 min. Consecutively, the uncaptured MCF-7 cells in solution were counted again by hemocytometry. The capture efficiency was defined as the ratio of the number of target cells captured to the number of target cells initially introduced. To release MCF-7 cells, photoresponsive immunomagnetic beads are treated by light photolysis at 365 nm, (10 mW/cm^2, 15 min) or 800 nm (10 mW/cm^2, 2 h). After light irradiation treatments, the photoresponsive immunomagnetic beads detached from MCF-7 cells were removed by magnetic scaffold and MCF-7 cells remained in solution were counted again to obtain release efficiency. To test the viability of the cells released by the light irradiation treatments, the released cells were collected in a new cell culture flask, with a total volume of 5 mL culture medium. Then the cells were put into the incubator for propagation in culture.

Toxicity of Materials and the Viability of Released Cells. The cytotoxicity of photoresponsive molecule was measured using the 3-(4,5)-dimethylthiahiazo(-z-y1)-3,5-diphenytetrazoliumromide (MTT) assay in breast cancer cell line MCF-7. Briefly, cells growing in log phase were seeded into 96-well cell-culture plate at 1×10^4 $well^{-1}$. The cells were incubated for 24 h at 37 °C under 5 % CO_2. The photoresponsive molecule compound 7 (Figure S2) at proper concentration were added to the wells of the treatment group, and 100 μL per well DMSO diluted in RPMI DMEM at final concentration of 0.2 % was added to the negative control group, respectively. (1) The description for "(Cell + photolinker)" means that cells were incubated with the compound 7 for 24 h at 37 °C under 5 % CO_2. (2) The description for "(Cell + photolinker +365 nm 10 mW/cm^2)" means that cells were first exposed with 365 nm light irradiation for 15 min and then incubated with the compound 7 for 24 h at 37 °C under 5 % CO_2. (3) The description for "(Cell + photolinker +800 nm 10 mW/cm^2)" means that cells were first exposed with 800 nm light irradiation for 2 h and then incubated with the compound 7 for 24 h at 37 °C under 5 % CO_2.

Calcein AM and propidium iodide (PI) were used to stain the released tumor cells to analyze their viability. Briefly, the cells were stained with 2 μM Calcein AM and 4 μM PI at room temperature for 30 min. Then the cells were observed under a fluorescence microscope. Further, the released MCF-7 cells were cultured under CO_2 incubator to detect their proliferation ability.

Capture and ICC Identification of Spiked Cancer Cells from Mimic Clinical Blood Samples. The healthy human whole blood was obtained from Renmin Hospital of Wuhan University. Mimic clinical samples were prepared by spiking MCF-7 cells into healthy human blood at 10^2 cells mL^{-1}. Then 0.2 mg anti-EpCAM-IgG-BPM-SA-MBs were added to the blood samples, and the mixture was incubated for 30 min at 37 °C (in the dark condition). The captured cells can be used for

common three-color immunocytochemistry (ICC) that cells were fixed with 4 % paraformaldehyde (30 min), permeabilized with 0.2 % Triton-X 100 (30 min), and stained with 10 μg / mL DAPI, FITC-labelled anti-CK19 monoclonal antibody, and PE-labelled anti-CD45 monoclonal antibody (following the kit instruction) for 2 h at 4 °C. After washing, the captured cells were put into a 96-well tissue culture plate for fluorescent microscopy imaging. The purity was calculated by dividing the number of cells captured by the total number of nucleated cells (DAPI+) in the samples.

Isolation of CTCs from Cancer Patients Blood Samples. Blood samples from 13 cancer patients and 8 healthy normal controls were collected and treated with anti-EpCAM-IgG-BPM-SA-MBs. Typically in each assay, a certain amount of blood (1 - 1.5 mL) was incubated with 0.2 mg anti-EpCAM-IgG-BPM-SA-MBs for 30 min (in the dark condition). After magnetic separation, the captured cells were identified with the three-color ICC and observed by a fluorescence microscope. Only the cells with phenotypes of DAPI+/CK+/CD45-, and with the appropriate size and morphology were counted as CTCs.

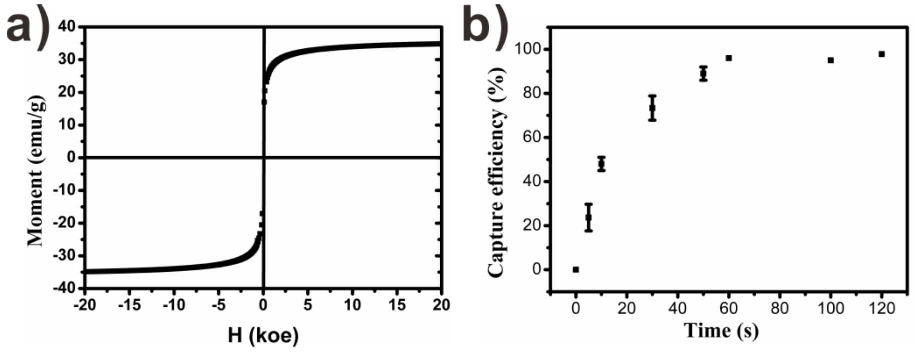

Figure S1. (a) Magnetic hysteresis loop of the MBs measured at room temperature. (b) Capture efficiencies of MBs at different attraction times with a commercial magnetic scaffold.

Photoresponsive molecular was synthesized using a method reported previously.[1-2]

Preparation of the compounds 2~7

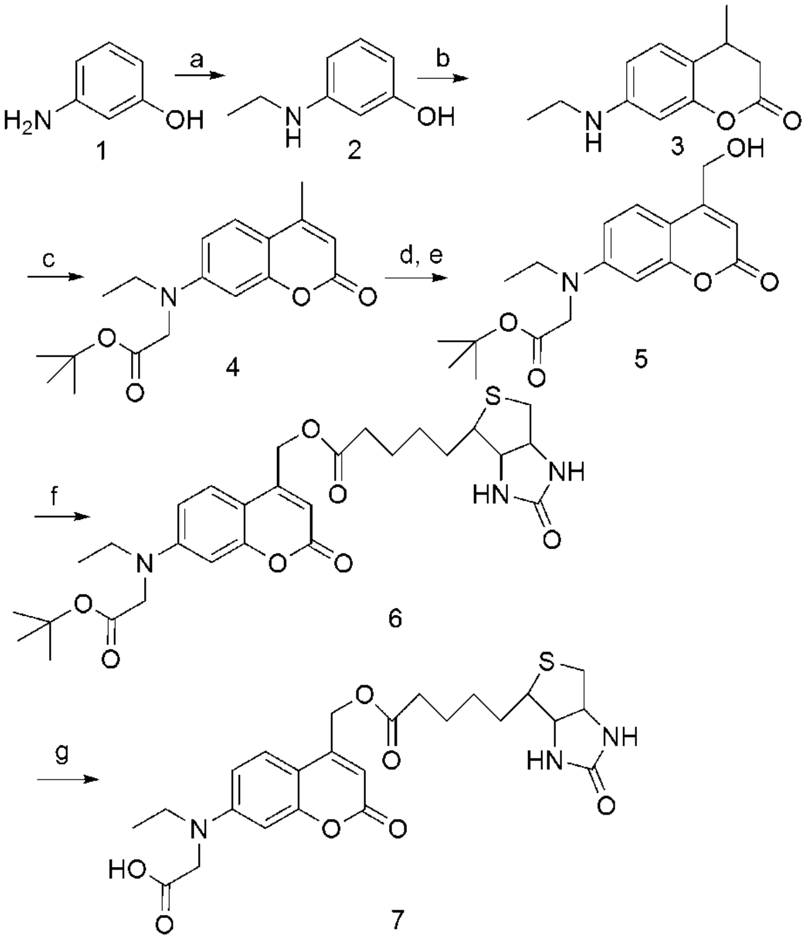

Figure S2. Synthetic scheme for preparation of compound 7. Reagents and conditions. (a) $ICH_2CH_3/K_2CO_3/KI$, DMF, 100 ºC, 2 h, 48 %; (b) $CH_3COCH_2COOEt/BiCl_3$, 75 ºC, 2d; 33 %; (c) $BrCH_2COOC(CH_3)_3/K_2CO_3/KI/TBAB$, CH_3CN, reflux, 4 d, 80 %; (d) SeO_2, p-xylene, reflux, 24 h; (e) $NaBH_4$, MeOH, rt, 3 h, 67 %; (f) biotin/EDC/DMAP, CH_2Cl_2, rt in the dark, 6h, 94 %; (g) TFA, CH_2Cl_2, rt, in the dark, 4 h;

Compound 2: A solution of 3-aminophenol (10 g, 92.5 mmol) and K_2CO_3 (12.5 g, 92.5 mmol) in DMF (15 mL) was stirred at 25 ºC for 15 min. Iodoethane (14 g, 92.5 mmol) was then added and heated at 100 ºC for 2 h. The reaction mixture was cooled to 25 ºC and filtered to remove solid

impurities. Water (30 mL) was added to the filtrate and extracted with EtOAc (20 mL × 3), then the combined organic layer was concentrated under reduced pressure, brown oil appeared. Purification with column chromatography (n-Hexane/EtOAc = 5/1) gave 5.9 g (45 mmol, 48 % yield). ^{1}H NMR ($CDCl_3$, 300 MHz): δ=7.01 (t, J=8.1 Hz, 1H, ArH), 6.18 (t, J=9Hz, 2H, ArH), 6.10 (s, 1H, ArH), 3.12 (q, J=7.5Hz, 2H, $-CH_2-$), 1.24 (t, J=7Hz, 3H, $-CH_3$); ^{13}C NMR ($CDCl_3$, 300 MHz): δ=157.33, 150.03, 130.81, 107.47, 106.36, 101.90, 39.48, 14.81; MS(ESI): 138.0$[M+H]^+$.

Compound 3: A mixture of 2 (3.1 g, 22.6 mmol), ethyl acetoacetate (3.2 mL, 24.9 mmol), and $BiCl_3$ (0.72 g, 2.3 mmol) was stirred at 75 °C for 2 days. The reaction mixture was then cooled to room temperature, diluted with dichloromethane and filtered. The obtained filtrate was evaporated under vacuum to yield brown oil. Purification with column chromatography (n-Hexane/EtOAc = 5/1) gave 1.5 g (7.5 mmol, 33 % yield) of 3 as a yellowish solid. ^{1}H NMR ($CDCl_3$, 300 MHz): δ=7.35(d, J=8.7Hz, 1H, ArH), 6.49(d, J=9Hz, 1H, ArH), 6.44(d, J=1Hz, 1H, ArH), 5.98(s, 1H, ArH), 3.22(t, J=6Hz, 2H, $-CH_2-$), 2.34(s, 3H, $-CH_3$), 1.30(t, J=6Hz, 3H, $-CH_3$); ^{13}C NMR ($CDCl_3$, 300 MHz): δ=162.29, 156.01, 153.37, 151.82, 125.55, 110.39, 109.01, 97.81, 38.08, 18.63, 14.47; MS(ESI):203.9$[M-H]^+$.

Compound 4: A solution of 3 (0.5 g, 2.5 mmol), tert-butyl bromoacetate (0.6 mL, 3.8 mmol), K_2CO_3 (1.0 g, 7.5 mmol), KI (38.0 mg, 0.25 mmol), and tetrabutylammonium bromide (81.0 mg, 0.25 mmol) in CH_3CN (20 mL) was refluxed for 3 days. The reaction mixture was allowed to cool to room temperature. After filtration, the solvent was removed under vacuum. The residue was then dissolved in CH_2Cl_2 and washed with H_2O (3 × 50 mL). The organic layer was then dried over

Na_2SO_4 and evaporated to yield the crude product. Purification with column chromatography (n-Hexane/EtOAc = 5/1) gave 0.63 g (2.0 mmol, 80 % yield) of 4 as yellow solid. ^1H NMR ($CDCl_3$, 300 MHz): δ=7.40 (d, J=9Hz, 1H, ArH), 6.55 (d, J=9Hz, 1H, ArH), 6.47(s, 1H, ArH), 5.99(s, 1H, RC=CH), 3.97(s, 2H, $-CH_2-$), 3.51(q, J=5.7Hz, 2H, $-CH_2-$), 2.35(s, 3H, $-CH_3$), 1.61(s, 9H, $-CH_3$), 1.25(t, J=7.2Hz, 3H, $-CH_3$); ^{13}C NMR ($CDCl_3$, 300 MHz): δ=169.41, 162.24, 155.82, 153.04, 150.96, 125.68, 110.26, 109.64, 108.72, 98.36, 82.36, 53.15, 46.87, 28.19, 18.64, 12.48; MS(ESI): 317.8[M^+].

Compound 5: SeO_2 (0.43 g, 2.8 mmol) and 4 (0.6 g, 1.9 mmol) were suspended in 50 mL p-xylene, the reaction mixture was refluxed under vigorous stirring with the protection of an argon atmosphere. After 24 h, the mixture was filtered and concentrated under reduced pressure. The dark brown residual oil was dissolved in methanol (50 mL), then sodium borohydride (0.14 g, 3.8 mmol) was added, and the solution was stirred for 3h at room temperature. Thereafter, the suspension was carefully neutralized with 1 M HCl, diluted with H_2O, and partially concentrated under reduced pressure to remove methanol. The mixture was extracted with CH_2Cl_2 and the obtained organic phase was washed with H_2O and brine, dried over Na_2SO_4, and concentrated in vacuum. The obtained oil was purified by column chromatography (n-Hexane/EtOAc= 5/2) to yield 0.40 g (1.27 mmol, 67 %) of 5 as a brown solid. ^1H NMR ($CDCl_3$, 300 MHz): δ=7.30(d, J=9.5Hz, 1H, ArH), 6.50(dd, J=9Hz, 2H, ArH), 6.31(s, 1H, RC=CH), 4.85(d, J=3Hz, 2H, $-CH_2-$), 3.97(s, 2H, $-CH_2-$), 3.50(q, J=7Hz, 2H, $-CH_2-$), 1.47(s, 9H, $-CH_3$), 1.24(t, J=9Hz, 3H, $-CH_3$). ^{13}C NMR ($CDCl_3$, 300 MHz): δ=169.73, 163.05, 156.00, 155.49, 150.76, 124.29, 108.90 107.39, 105.57, 98.08, 82.48, 60.27, 52.88, 46.64, 28.07, 12.32; MS(ESI): 333.8[M^+].

Compound 6: In dark condition, a mixture of 5 (500 mg, 1.59 mmol), biotin (284 mg, 1.17 mmol) EDC (372 mg, 1.94 mmol), DMAP (12 mg, 0.097 mmol) was dissolved in 40ml anhydrous CH_2Cl_2 under an argon atmosphere. After stirring at RT for 6 h, the solvent was removed under vacuum. The obtained crude product was purified by column chromatography (EtOAc : MeOH = 100 : 3~5) to give 530 mg (0.82 mmol, 94 % yield) of 6 as a yellowish solid. ^{1}H NMR ($CDCl_3$, 300 MHz): δ=7.40(d, J=9Hz, 1H, ArH), 6.65(m, 1H, ArH), 6,52(s, 1H, ArH), 6.37(s, 1H, RC=CH-), 4.67(s, 2H, -CH_2-), 4.53(m, 2H, R_3CH), 4.30(t, J=7.2Hz, 2H, -CH_2-), 3.37(s, 2H, -CH_2-), 3.30(m, 1H, R_3CH), 2.28(d, J=3.6Hz, 2H, -CH_2-), 1.70(m, 2H, -CH_2-), 1.65(m, 2H, -CH_2-), 1.52(s, 9H, -CH_3), 1.32(t, J=3.6Hz, 2H, -CH_2-), 1,23(t, J=3Hz, 2H, -CH_2-). ^{13}C NMR ($CDCl_3$, 300 MHz): δ=173.15, 169.48, 164.75, 160.81, 155.46, 149.17, 147.30, 125.12, 111.75, 110.68, 108.56, 97.16, 81.82, 62.65, 61.73, 60.8, 58.3, 55.65, 44.37, 41.1, 34.9, 29.08, 28.63, 24,83, 12.65; MS(ESI): 560.3$[M+H]^+$.

Compound 7: In dark condition, to a solution of tert-butyl ester 6 (300mg, 0.46mmol) in CH_2Cl_2 (3ml) was added TFA (5 ml) dropwise. The reaction mixture was stirred for 4 h at room temperature (In dark condition). After removing the solvent, TFA and by-products under vacuum, the obtained yellow solid was then washed by water several times and dried under vacuum. The gained compound was directly used in the next step without purification, as the reaction was efficient, and the target compound was of extremely polar and unable to desorb from the silica gel. MS (ESI): 502.2$[M-H]^+$.

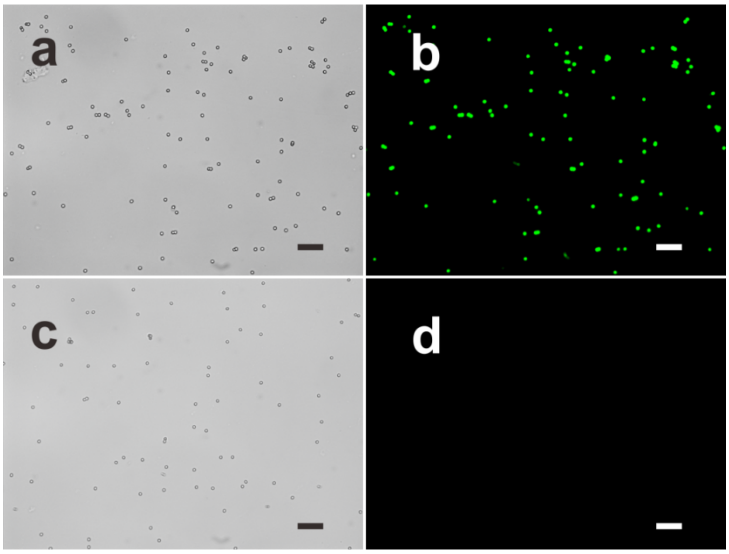

Figure S3. (A, B) Incubation of FITC-biotin with SA-MBs; (C, D) Incubation of FITC-biotin with MBs; All scale bars represent 10 μm.

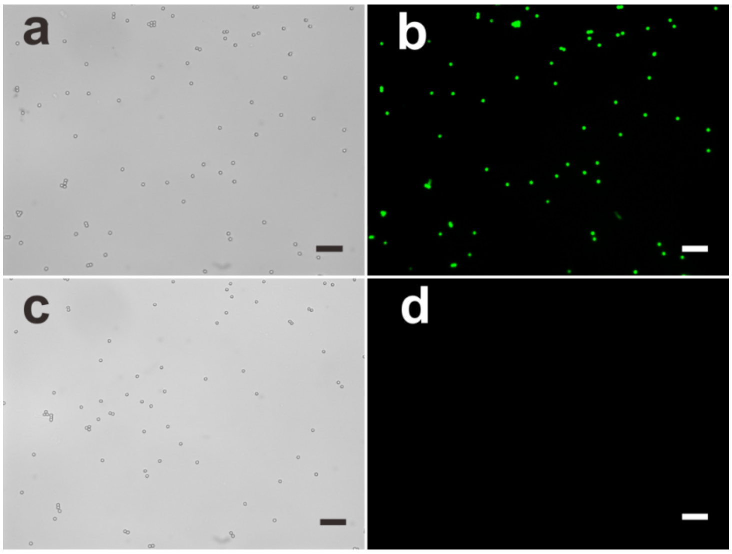

Figure S4. (A, B) Incubation of FITC-labeled rabbit anti-goat antibody with IgG-BPM-SA-MBs; (C, D) Incubation of FITC-labeled rabbit anti-goat antibody with BPM-SA-MBs; All scale bars represent 10 μm.

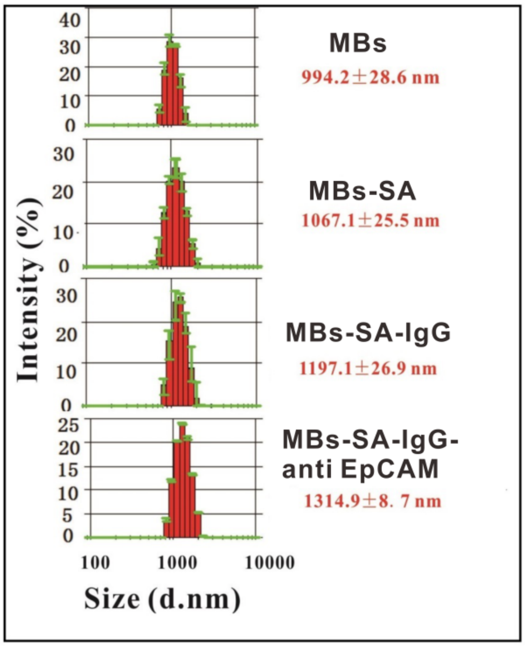

Figure S5. Sequential size distribution detection of the construction of photoresponsive immunomagnetic beads.

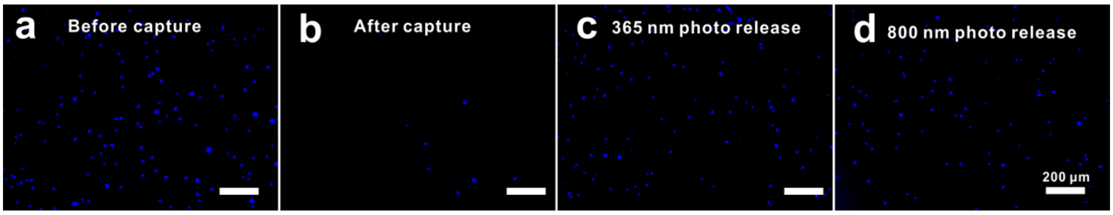

Figure S6. Photochemical release of the captured MCF-7 cells from an anti-EpCAM-IgG-BPM-SA-MBs. (a) before cell capture, (b) after cell capture, and (c) after 365 nm photon release. An average of 73 ± 4 % of the captured MCF-7 cells can be released at 365 nm light irradiation (10 mW/cm^2) for 15 min. (d) after NIR 800 nm release. An average of 52 ± 6 % of the captured MCF-7 cells can be released at 800 nm light irradiation (10 mW/cm^2) for 2 h.

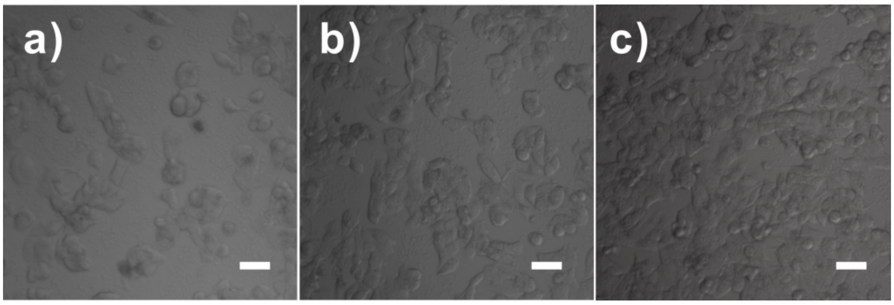

Figure S7. Viability analyses of the isolated tumor cells. Proliferation of the released MCF-7 cells is depicted (a) seeding after release. (b) reached confluence after one passage. (c) subpassage.

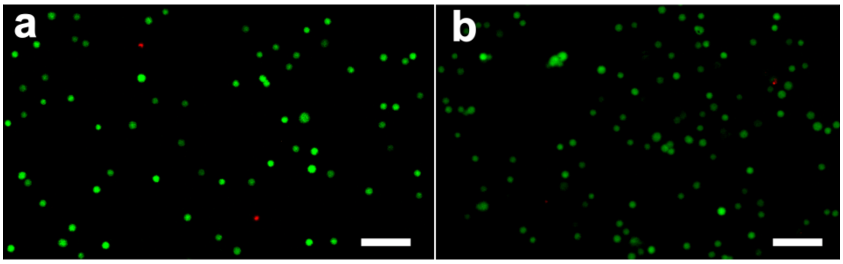

Figure S8. Viability analyses of the released tumor cells. (a) Fluorescence microscopic image of the released cells (365 nm 10 mW/cm^2)stained with calcein AM (green) and PI (red).(B) Fluorescence microscopic image of the released cells (800 nm 10 mW/cm^2) stained with calcein AM (green) and PI (red).

Table S1. The interplay between irradiation time and cell viability.

wavelength and dose	Time/min	Mean cell viability/%
	10	94
365 nm	15	90
10 mW/cm^2	20	83
	30	70
	60	26

Table S2. Quantification of CTCs of blood samples from patients with epithelial cancers.

Patient ID	Cancer Type	Gender	Age	Volume Sample/ml	CTCs
1	Lung	M	65	1	9
2	Lung	M	55	1	136
3	Lung	M	57	1	37
4	Lung	M	65	1.3	203
5	Lung	M	59	1	23
6	Lung	M	82	1.5	10
7	esophagus	M	73	1.3	7
8	Colon	F	66	1	17
9	liver	M	63	1.1	2
10	breast	F	42	1	4
11	gastric	M	49	1	1
12	gastric	F	44	1	11
13	gastric	M	73	1	5

Table S3. Quantification of circulating tumor cells per mL of blood in healthy subjects. None of the samples from healthy subjects had any detectable CTCs.

Healthy Sample ID	Gender	Age	Volume Sample/ml	CTCs
1	M	49	1	0
2	F	25	1	0
3	M	34	1	0
4	M	25	1	0
5	F	29	1	0
6	M	51	1.3	0
7	F	51	1	0
8	F	48	1.1	0

The mechanisms of photo-induced release

The coumarinylmethy moieties are members of arylalkyl-type photo-removable protecting groups and photolysis of their caged derivatives with the presence of water produces cleavage of a C-O bond, forming magnetic beads and antibody.[3-5]

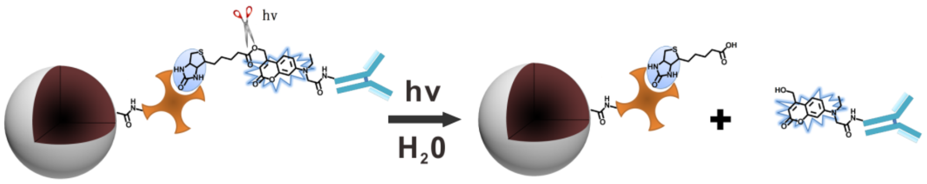

Reference:

1. Q. Lin, Q. Huang, C. Y. Li, C. Y. Bao, Z. Z. Liu, F. Y. Li, L. Y. Zhu, *J. Am. Chem. Soc.* 2010, **132**, 10645–10647.

2. Q. Lin, C. Y. Bao, S. Y. Cheng, Y. L. Yang, W. Ji, L. Y. Zhu, *J. Am. Chem. Soc*. 2012, **134**, 5052 –5055.

3. V. Hagen, B. Dekowski, N. Kotzur, R. Lechler, B. Wiesner, B. Briand, M. Beyermann, *Chem. Eur.*

J. 2008, **14**, 1621-1627.

4. A. Z. Suzuki, T. Watanabe, M. Kawamoto, K. Nishiyama, H. Yamashita, M. Ishii, M. Iwamura, T. Furuta, *Org. Lett*. 2003, **5**, 4867.

5. T. Furuta, T. Watanabe, S. Tanabe, J. Sakyo, C. Matsuba, *Org. Lett*. 2007, **9** 4717.
